# Hyperglycemia in Turner syndrome: Impact, mechanisms, and areas for future research

**DOI:** 10.3389/fendo.2023.1116889

**Published:** 2023-02-15

**Authors:** Cameron Mitsch, Eirene Alexandrou, Andrew W. Norris, Catherina T. Pinnaro

**Affiliations:** ^1^ Department of Health and Human Physiology, The University of Iowa, Iowa City, IA, United States; ^2^ Stead Family Department of Pediatrics, University of Iowa, Iowa City, IA, United States; ^3^ Fraternal Order of Eagles Diabetes Research Center, University of Iowa, Iowa City, IA, United States

**Keywords:** Turner syndrome - TS, diabetes mellitus, impaired glucose tolerance, growth hormone, estrogen, X chromosome (human)

## Abstract

Turner syndrome (TS) is a common chromosomal disorder resulting from complete or partial absence of the second sex chromosome. Hyperglycemia, ranging from impaired glucose tolerance (IGT) to diabetes mellitus (DM), is common in TS. DM in individuals with TS is associated with an 11-fold excess in mortality. The reasons for the high prevalence of hyperglycemia in TS are not well understood even though this aspect of TS was initially reported almost 60 years ago. Karyotype, as a proxy for X chromosome (X_chr_) gene dosage, has been associated with DM risk in TS – however, no specific X_chr_ genes or loci have been implicated in the TS hyperglycemia phenotype. The molecular genetic study of TS-related phenotypes is hampered by inability to design analyses based on familial segregation, as TS is a non-heritable genetic disorder. Mechanistic studies are confounded by a lack of adequate TS animal models, small and heterogenous study populations, and the use of medications that alter carbohydrate metabolism in the management of TS. This review summarizes and assesses existing data related to the physiological and genetic mechanisms hypothesized to underlie hyperglycemia in TS, concluding that insulin deficiency is an early defect intrinsic to TS that results in hyperglycemia. Diagnostic criteria and therapeutic options for treatment of hyperglycemia in TS are presented, while emphasizing the pitfalls and complexities of studying glucose metabolism and diagnosing hyperglycemia in the TS population.

## 1 Introduction

Turner syndrome (TS) is a common, non-heritable genetic disorder affecting ~1 in 2000 females and is caused by complete or partial absence of the second sex chromosome (1). Most individuals with TS have short stature and primary ovarian insufficiency, which require timely treatment with recombinant growth hormone (GH), estrogen, and other adjuncts over prolonged periods to facilitate linear growth and induce and maintain puberty. Other associated features of TS may include lymphedema, specific cognitive traits or deficits, congenital heart disease, autoimmunity, osteoporosis, renal malformations, and diabetes mellitus (DM) (2). This review will focus on the TS hyperglycemia phenotype – an indolent and gradually progressive decline in oral glucose tolerance that often culminates in frank DM (3). Despite the prevalence of hyperglycemia in TS, the pathogenesis has not been clarified, and as a result, no specific prevention or treatment exists. This review will cover the epidemiology and impact of hyperglycemia on those with TS, the hypothesized genetic and physiologic mechanisms associated with hyperglycemia in TS, and diagnostic and therapeutic approaches, while emphasizing the challenges of studying glucose metabolism in the TS population.

## 2 Search methods

**Figure 1:**
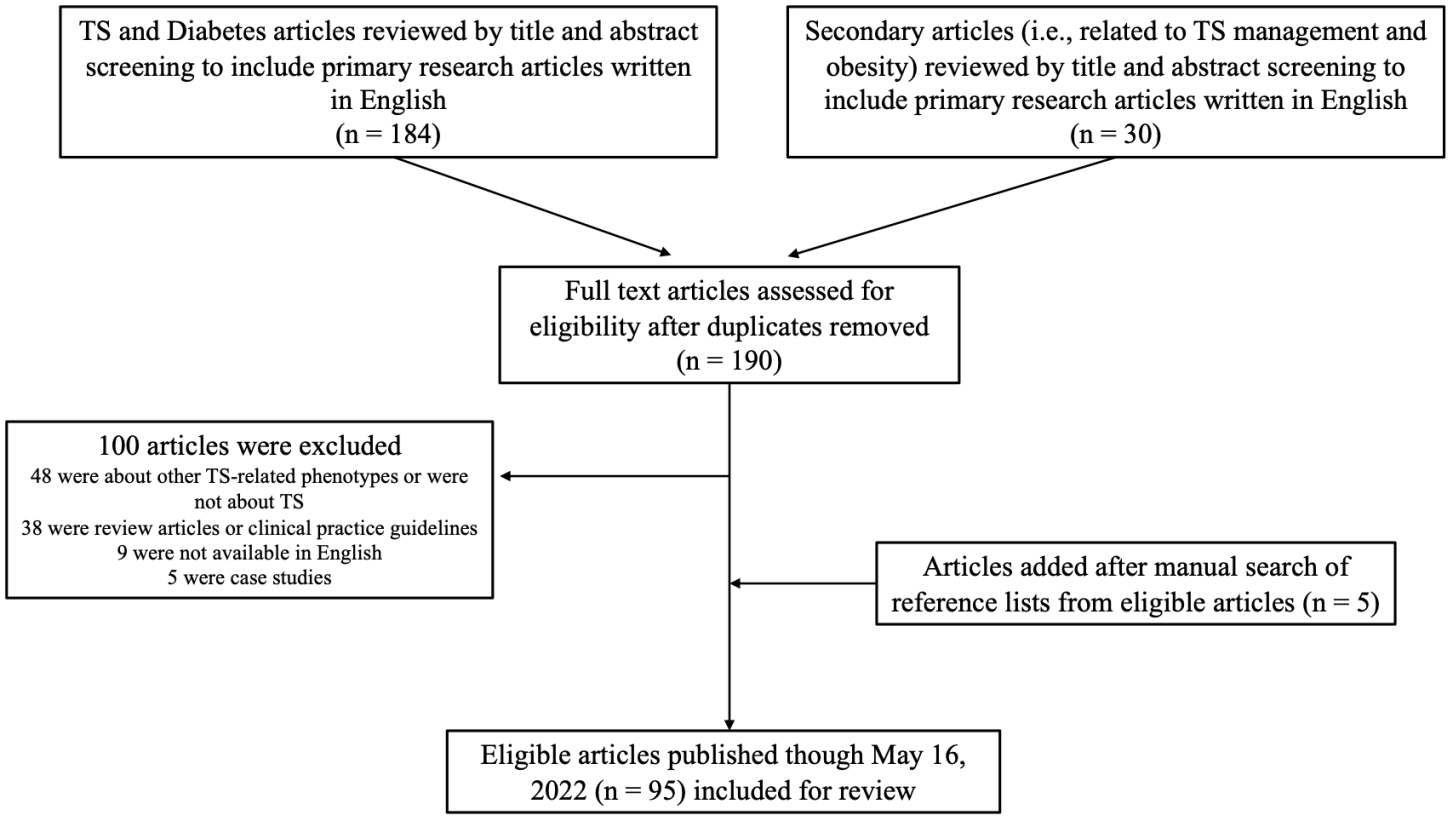
Flow chart of literature search.

A comprehensive literature search was conducted using PubMed (https://pubmed.ncbi.nlm.nih.gov) to retrieve all relevant primary research articles related to glucose metabolism in TS published in English through May 16, 2022, producing 184 articles. The search was carried out using the following keywords, either alone or in combination using the Boolean method: [(“Turner Syndrome” OR “monosomy X”) AND (diabetes mellitus OR “impaired glucose tolerance” OR hyperglycemia)]. We additionally performed searches to identify articles related to confounders of the study of hyperglycemia in TS, including obesity in TS and the impact of TS treatments on glucose metabolism, specifically recombinant growth hormone (GH), estrogen, and oxandrolone. We identified 190 unique articles after assessing for duplicates. The full text of these articles was reviewed and narrowed to 90 articles meeting eligibility criteria. We subsequently performed a manual search of the reference lists of all 90 articles and identified 5 additional eligible articles. The cumulative review consisted of 95 articles. A flow chart of our literature review methods is depicted in **Figure 1**.

## 3 Epidemiology/scope

Individuals with TS have a higher risk of mortality at all ages (4–6), and DM is associated with a further 11-fold raised mortality risk in TS (4). The high incidence of hyperglycemia in TS was noted over 50 years ago (7), but the true epidemiologic prevalence of hyperglycemia in TS remains difficult to estimate, partly because of selection bias in small, cross-sectional studies. Additionally, there is a lack of standardization in assessing glucose tolerance in TS patients, with studies reporting results using 50-, 75-, and 100-gram oral glucose tolerance tests (OGTT). Compounding the problem, these studies use varied diagnostic criteria, including those provided by the American Diabetes Association/National Diabetes Database (8, 9), Joslin (10), the United States Public Health Service (11), the World Health Organization (12), the Canadian Diabetes Association (13), and non-standard criteria (14–17). The prevalence of hyperglycemia in TS-specific studies using OGTT (assessed as described) is up to 83%, with higher rates reported in studies that enrolled older participants (3, 14, 18). However, asymptomatic hyperglycemia is not limited to adults and has been observed in up to 40% of children with TS aged 5 to 12 years old (16).

Several treatment modalities used in the management of TS have the potential to alter glucose metabolism, especially recombinant GH, estradiol, and oxandrolone (2). Supraphysiologic levels of GH, as demonstrated in patients with acromegaly, lead to increased liver gluconeogenesis, as well as hepatic and peripheral insulin resistance (19). Likewise, insulin resistance is known to worsen in puberty for karyotypically-normal individuals, in healthy young women treated with oral contraceptive therapies, and in women with hyperandrogenemia (20, 21). However, both treatment-naïve and hormonally treated children and adults with TS show altered glucose metabolism (22), and the high prevalence of hyperglycemia in TS was noted prior to the introduction of hormonal therapies as components of TS management. Thus, hyperglycemia in TS cannot solely be deemed iatrogenic.

## 4 Effects of TS management on glucose homeostasis

### 4.1 Recombinant growth hormone

Recombinant GH at supraphysiologic doses (0.045-0.050 mg/kg/day, up to 0.068 mg/kg/day) is recommended in TS, typically starting around 4-6 years old and ideally, before age 13 years (2). Growth promoting treatments are utilized until growth plate fusion. Several studies have examined the influences of recombinant GH on glucose metabolism during this extended treatment period and upon its discontinuation. Overall, studies have demonstrated a worsening of insulin resistance without increased prevalence of IGT or DM in TS patients treated with recombinant GH (19, 22–24). A dose-related increase in insulin resistance was noted in patients treated with higher doses of recombinant GH (0.045 mg/kg/day *vs* 0.0675 mg/kg/day and 0.09 mg/kg/day), as evidenced by increased area under the curve (AUC) for insulin during OGTT, insulinogenic index, and urinary C-peptide (24). There was no apparent difference in glucose metabolism in once daily versus twice daily divided doses of recombinant GH. Additionally, fasting insulin showed a sustained increase throughout GH therapy, in contrast to glucose-stimulated insulin, which showed no progressive worsening beyond four years of treatment (19, 22). Finally, insulin resistance induced by recombinant GH appeared to be reversible, as insulin levels declined upon discontinuation of treatment, although remained above pre-treatment levels (19, 22, 23). Studies involving non-TS controls also saw increased insulin levels in 46, XX females; therefore, the elevated post-treatment insulin levels in TS individuals were felt to be positively correlated with post-pubertal status and increasing age (22, 23). Thus, while recombinant GH treatment is responsible for reversible increases in insulin resistance, it does not explain the lifetime increased risk of hyperglycemia in TS.

### 4.2 Sex hormone replacement

Studies examining the effect of hormone replacement therapy (HRT) on glucose metabolism in TS demonstrate improvements in insulin sensitivity. Limited published studies have evaluated their effect on glucose tolerance. An investigation of the impact of six months of HRT with either oral or transdermal 17-beta-estradiol plus norethisterone in adult TS patients compared to healthy controls demonstrated that HRT did not worsen insulin resistance, but the AUC glucose on OGTT significantly increased (20). These differences were comparable between the transdermal versus oral estradiol groups, demonstrating no perceived benefit of one therapy over the other with regards to carbohydrate metabolism. A subsequent comparison of transdermal versus oral estradiol replacement on metabolic parameters in women with TS similarly did not demonstrate a perceived metabolic benefit of one therapy over the other although this study only measured fasting glucose (25). Additionally, HRT ameliorated the recombinant GH-induced increase in insulin resistance in women with TS (26). Another study also demonstrated that the GH-induced increase in insulin resistance was improved by the addition of estradiol; however, estradiol had no effect on glucose tolerance (27). Thus, HRT in TS seems to improve insulin sensitivity, but the effect on glucose tolerance (either alone or in conjunction with recombinant GH or alone) is not clear but is likely minor.

### 4.3 Oxandrolone

Oxandrolone is a non-aromatizable androgen utilized for adjunctive growth promotion in TS (28). Data regarding oxandrolone’s effect on glucose metabolism are mixed. A randomized, placebo-controlled study comparing recombinant GH in combination with placebo or oxandrolone (either at 0.03 or 0.06 mg/kg/day), demonstrated no significant impact on insulin sensitivity (29). There were no differences between (recombinant GH + oxandrolone) versus (GH + placebo) groups with regards to developing IGT or decreased insulin sensitivity; however, fasting glucose levels and hemoglobin A1c (HbA1c) values decreased more in those treated with oxandrolone compared with placebo. In contrast, some studies have demonstrated a worsening of glucose tolerance with oxandrolone (21, 30). Specifically, AUC glucose and insulin levels during OGTT rose in patients treated with oxandrolone, either alone or in combination with recombinant GH (21). Despite these changes, HbA1c levels and fasting glucoses in treated individuals remained normal. This study used oxandrolone doses higher (0.125 mg/kg/day) than current recommendations (<0.05 mg/kg/day) (2). Similar increases in AUC insulin levels were noted during OGTT in individuals with TS on GH/oxandrolone combination therapy in a subsequent study, which also demonstrated a reversibility of glucose intolerance in TS individuals upon discontinuation of these treatments (30).

In summary, the effects of oxandrolone and estrogen on glucose metabolism are less clear than the effect of recombinant GH, but all may impact glycemic response as well as insulin resistance. Accordingly, recombinant GH, estrogen, and oxandrolone should be noted and controlled for when designing and interpreting clinical studies evaluating glucose metabolism in individuals with TS.

## 5 Clinical and genetic factors involved in hyperglycemia in TS

### 5.1 Non-TS-specific

#### 5.1.1 Age

The incidence and prevalence of type 2 DM in the general population increases with age (31). Both cross-sectional (32) and prospective, longitudinal studies (3, 33) show that, not surprisingly, the risk of hyperglycemia also increases with age in TS. This phenomenon persists independent of current or prior recombinant GH use (33), and current (33, 34) or prior (34) estrogen-replacement therapy. Age-related risk of hyperglycemia was also noted in a study of children and adolescents who had never received hormone replacement of any kind (16).

#### 5.1.2 Obesity

Obesity is also a known risk factor for metabolic syndrome and type 2 DM in the general population, and obesity is described as a common comorbidity of TS. However, there is no standard definition of obesity in TS. BMI correlates with several biochemical markers of obesity in TS, including C-reactive protein and interleukin-6 (35) and is the most assessed proxy of adiposity in clinical studies. However, when individuals with TS were matched to 46, XX women based on BMI, the women with TS demonstrated excess visceral and internal abdominal adipose tissue and intrahepatocellular lipids on magnetic resonance imaging (36). Two follow-up studies showed that alternative methods more specific to visceral fat may better represent metabolic risk in TS (37, 38). Body weights of those with TS are often greater than those of 46, XX girls and women of the same height (39), even though individuals with TS tend to have less subcutaneous extremity fat (39, 40). Those with TS have increased total fat mass and visceral fat with decreased total lean body mass (41, 42). For these reasons, it is plausible that typical BMI percentiles underestimate metabolic risk in individuals with TS.

Even with the aforementioned caveats, BMI is the most widely reported measure of obesity in glucose metabolism studies in TS. Most studies support linkage of increasing BMI with hyperglycemia in TS, but the risk is not obligatorily coupled to obesity. In a cross-sectional study consisting of children and adolescents with TS and controls, there was no difference in weight excess (reported as BMI-standard deviation score) in those with TS who had abnormal OGTTs compared to the 31 individuals (those with TS and controls) who had normal OGTTs (16). In contrast, a large longitudinal study comprised of 113 TS patients demonstrated that BMI was higher among those with TS who had IGT compared to those with normal glucose tolerance (NGT) (3). BMI percentiles were also positively correlated with metabolic comorbidities including HbA1c in a recent natural history study of TS (43). Additionally, increasing BMI was associated with the development of hyperglycemia among those with TS who were not considered obese by BMI (32), and a longitudinal study (of a different group of individuals with TS) demonstrated a positive correlation of BMI with fasting glucose and HbA1c (43). Thus, it appears that within TS cohorts, increasing BMI is associated with increased risk of hyperglycemia even at non-obese BMI.

#### 5.1.3 Family history of diabetes mellitus

An early observation noted in the study of TS was a high prevalence of DM and autoimmunity in the parents of individuals with TS (7). A second small study supported this observation (44). It was thus hypothesized that parental hyperglycemia or autoimmunity may impair meiosis and increase the risk of sex chromosome abnormalities such as TS (45, 46). Despite the initial enthusiasm for this topic, many well-designed follow up studies–which included comparable control groups–failed to demonstrate an association of family history of DM with risk to have a child with TS (33, 34, 47). Consistent with this negative conclusion, a study of a large group of individuals with TS found that the prevalence of DM in first-degree relatives of individuals with TS was similar to that in the general population (48). However, the relationship between family history of DM and development of TS-associated hyperglycemia has not been thoroughly explored. Family history of type 2 DM is a strong risk factor for the development of type 2 DM in the general population and largely represents genetic risk (49), but the impact of family history on the development of DM in TS is limited as most studies do not collect family history information in the setting of glycemic phenotyping.

#### 5.1.4 Hypogonadism

There is both epidemiologic and experimental evidence to suggest that post-pubertal sex steroids contribute to sex-related differences in diabetes susceptibility. Specifically, the protective role of endogenous estrogens dissipates as women undergo menopause, evidenced by deleterious effects on body composition and glucose homeostasis (50) that can potentially be mitigated by estrogen replacement (51, 52). This is especially relevant to TS, as absent or incomplete pubertal development is one of the cardinal features (53), and pubertal induction and maintenance is a mainstay of TS treatment (2). Therefore, estrogen deficiency is a biologically plausible mechanism of increased diabetes incidence in TS. However, the increased incidence of hyperglycemia persists in individuals with TS who are actively on sex steroid treatment (18, 20, 54) and in pre-pubertal children with TS (16, 54). Thus, it is unlikely that estrogen deficiency alone underlies the increased risk of DM in TS. The effect of sex hormone replacement on carbohydrate metabolism in TS is reviewed in section 4.2 above.

### 5.2 TS-specific

#### 5.2.1 X chromosome genetics and risk of hyperglycemia in TS

In addition to estrogen, X chromosome (X_chr_) dosage has been shown to contribute to the favorable metabolic profile in 46, XX women (55, 56). Since TS is associated both with estrogen deficiency and haploinsufficiency for the X_chr_, the TS hyperglycemia phenotype could be related to X_chr_ genetic factors, estrogen deficiency secondary to gonadal insufficiency, or their interaction.

Multiple karyotypes, all which result in haploinsufficiency of X_chr_ genes, are associated with TS. These include 45X (complete absence of the second X_chr_), deletions of the short arm (i.e., p arm) of the second X_chr_, isochromosome Xq (i.e., the second X_chr_ is comprised of 2 q arms and no p arms, equating to deficiency of the p arms), ring X_chr_ (i.e., the second X_chr_ is a ring containing variable amounts of X_chr_ material) and other structural aberrations (i.e., unbalanced X_chr_-autosome translocations) (57). Any of these TS-associated karyotypes can exist with mosaicism for a typical 46XX, 46XY, 47XXX, or the aforementioned TS cell lines and may still present with features of TS depending on the prevalence and distribution of the of the TS cell line(s) (58).

Interestingly, even when compared to 46, XX women with primary ovarian insufficiency, women with TS demonstrate significant impairments in oral glucose-stimulated insulin secretion, which implicates X_chr_ factors, independent of estrogen deficiency, in the TS hyperglycemia phenotype (59). Karyotype, as a proxy for X_chr_ gene dosage, was independently associated with DM risk in TS, with isochromosome Xq demonstrating the greatest risk increase (32). This suggests that haploinsufficiency for Xp genes coupled with supernumerary copies of Xq genes (as is seen in isochromosome Xq) may underly the pathogenesis of the TS hyperglycemia phenotype. However, two subsequent studies reported that metabolic comorbidities, including hyperglycemia, are more highly associated with a different TS karyotype (i.e., ring X chromosomes) (58, 60). A strength of both these studies is the large number of TS individuals that were involved (>1000 each), while limitations include their observational, retrospective nature and their reliance on HbA1c to diagnose DM. Thus, there is no consensus on X_chr_ location of potential TS hyperglycemia-related genes.

X_chr_ inheritance in TS is biased, with approximately three-fourths of 45X TS individuals inheriting a maternal X_chr_ (61). This has led to the hypothesis that there may be X_chr_ genes which are differentially expressed depending on parent-of-origin. One recent study found no impact of X_chr_ parent-of-origin on fasting blood glucose in TS (62), but the lack of association is not surprising given that impaired fasting glucose is rare among those with TS (43), thus limiting statistical power in TS studies focused on this outcome. Another recent study also found no association of X_chr_ parent-of-origin with DM, but there were actually no individuals with DM in this study (63). Other metabolic phenotypes, including hypercholesterolemia (64, 65), are associated with parent-of-origin in TS, which suggests that there are imprinted genes on the X_chr_ that may underlie some of the TS-associated phenotypes.

**Figure 2:**
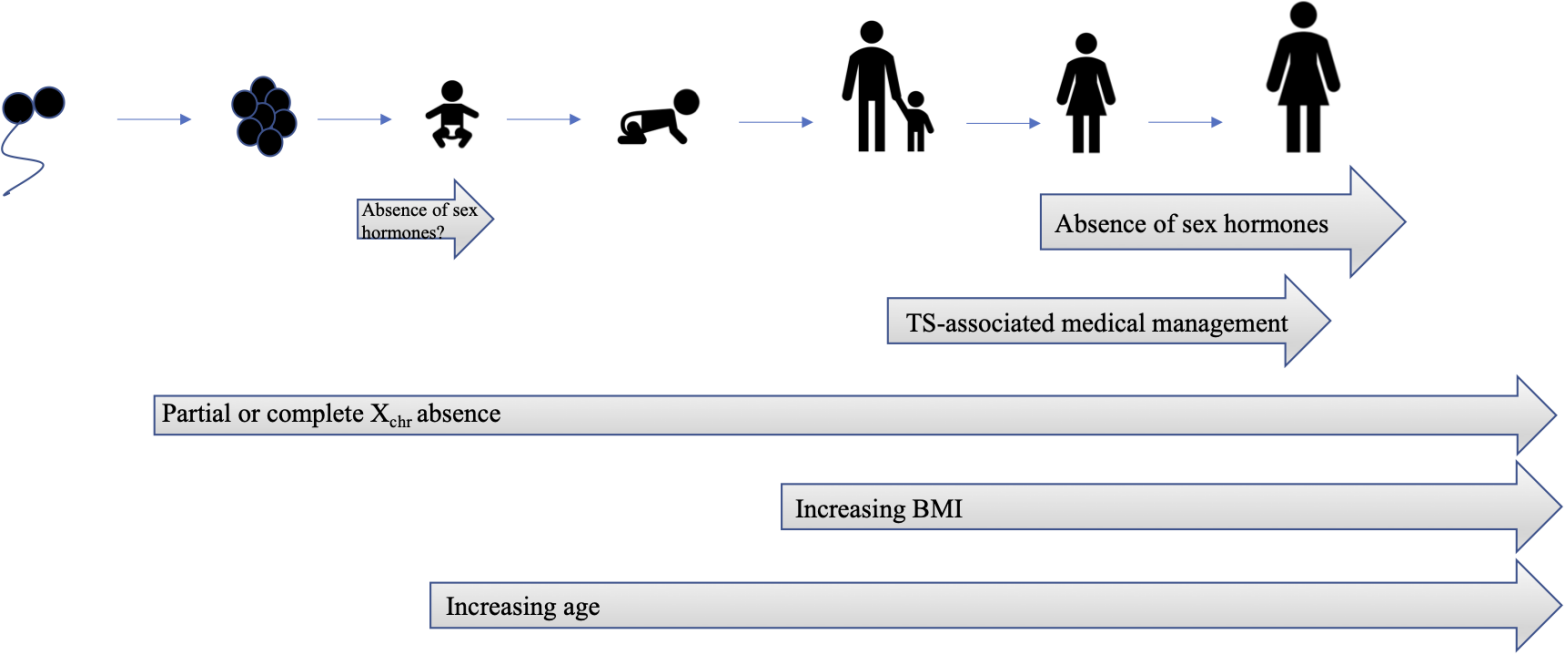
Evolution of risk factors for TS-associated hyperglycemia. There is an intrinsic risk of hyperglycemia due to syndrome-specific X_chr_ factors that is present at birth. Similar to type 2 DM in the general population, the risk for hyperglycemia increases with age and BMI. Temporary increases in insulin resistance are attributable to medical therapy, such as recombinant GH, and improve after discontinuation of therapy. Gonadal insufficiency and decreased production of sex hormones also contributes to risk of hyperglycemia, although is no.

Haploinsufficiency for X_chr_ genes leads to global hypomethylation and altered autosomal gene expression (66, 67). Several studies have demonstrated abnormal gene expression profiles (32, 68) and differentially methylated X_chr_ and autosomal genes in individuals with TS (68, 69) Functional annotations of TS datasets (RNA sequencing and DNA methylation analysis of fibroblasts) demonstrate enrichment in genes involved in carbohydrate metabolism and glucose import (68, 70). Two recent studies have employed bioinformatics approaches to identify differentially expressed genes in TS and used pathway analysis to determine key differentially regulated pathways relevant to diabetes (71, 72). Candidate signature genes of DM in TS include 8 upregulated autosomal genes, including *SLC29A2, THBS1, GPRC5B, CSHL1, ADAM22, IGHM, WIZ, IGHD*, and 1 downregulated autosomal gene, *COX11* (72). It is not known if these altered autosomal genetic signatures are involved in the pathogenesis of diabetes in TS individuals. Despite significant recent progress made in characterizing the TS genome and epigenome, no specific genes or loci have been implicated definitively in the TS hyperglycemia phenotype. **Figure 2** summarizes the evolution of risk factors for TS-associated hyperglycemia.

## 6 Physiological mechanisms hypothesized to underlie hyperglycemia in TS

Dysregulated secretion of GH, autoimmune beta cell destruction, impaired insulin secretion, increased insulin resistance, hyperglucagonemia, and an impaired incretin axis have all been hypothesized to be the primary or major physiological mechanism(s) contributing to hyperglycemia in TS. Many studies have drawn conclusions based on small sample sizes (median sample size 21). Early studies used hormonal assays that were hampered by a large degree of variability, which further complicates interpretation. Additionally, many studies were confounded by the absence of control groups matched by age, sex and/or adiposity, which are all variables known to impact glucose metabolism. Hypogonadal status and short stature make matching especially difficult in TS, given that women with TS may have increased adiposity that is differentially distributed anatomically compared to non-TS individuals (41, 42) as reviewed in section 5.2. Age of TS patients and TS management therapies should be accounted for (see sections 4 and 5.1 above for additional details) as potential confounders. Particular attention should be paid to studies including individuals with TS who are at or near the age of puberty– there is a well-described physiological decline in insulin sensitivity accompanied by compensatory insulin secretion that occurs during puberty (73), and it is unclear whether individuals with TS undergo this physiological transition, especially if not treated with sex steroids to facilitate pubertal induction and/or maintenance. Thus, comparing individuals with TS to age-matched controls during puberty may result in a biased conclusions of lower insulin secretion and decreased insulin resistance in TS. **Table 1** summarizes the TS-non-specific and TS-specific factors that may contribute to hyperglycemia risk in TS.

**Table 1:** Summary of therapeutic, clinical, and genetic factors thought to be related to increased risk of hyperglycemia in TS.

Potential Risk Factor for DM in TS	Increases risk of diabetes in TS?	Supporting data
Recombinant GH	No	Responsible for reversible increases in insulin resistance but its use does not explain the lifetime increased risk of hyperglycemia in TS
Sex Hormone Replacement	No	Improves insulin resistance, effect on glucose tolerance is not clear
Oxandrolone	No	Unclear, often utilized in conjunction with recombinant GH and estrogen replacement
Age	Yes	Young individuals are still noted to have hyperglycemia at higher rates than expected, but risk does increase with age.
Obesity	Yes	No standard definition of obesity in TS, but increasing BMI is associated with increased risk of hyperglycemia in TS even at non-obese BMI.
Family history of diabetes mellitus	Unknown	Studies evaluating family history of DM in TS were not specifically evaluating that relationship to risk of DM in TS, rather they sought to see if parental DM was a risk factor for having a child with TS. These studies do not include enough data related to DM phenotype to conclude whether family history increases the risk of DM in TS.
Hypogonadism	Yes	HRT does not eliminate increased risk of hyperglycemia in TS, so unlikely to be sole contributing risk factor.
Complete or partial absence of the second sex chromosome	Yes	When compared with an age, sex, and BMI-matched cohort of women with primary ovarian failure (thus removing estrogen deficiency as a confounder), the TS group had higher rates of glucose abnormalities and demonstrated impaired insulin secretion.

TS, Turner syndrome; DM, diabetes mellitus; GH, growth hormone; HRT, hormone replacement therapy; BMI, body mass index.

### 6.1 Dysregulated endogenous GH secretion

Early in the study of hyperglycemia of TS, excess circulating GH was documented in 4 individuals with TS (74). Thus, it was hypothesized that excess GH secretion was responsible for the hyperglycemia seen in TS, similar to acromegaly. It should be cautioned that the early study utilized the tibia line assay, which can give values which are much higher than those from radioimmune assays (14). Five additional studies followed up on the GH hypothesis (17, 33, 47, 75, 76). The studies involved between 14-30 individuals of varying ages with TS. Three studies measured fasting GH levels using various radioimmunoassay techniques (17, 47, 76), and in one study, the assay method is not described (75). No difference in fasting GH levels was noted between individuals with TS and normal glucose metabolism and those with TS and hyperglycemia (75), or between individuals with TS and non-sex-matched controls. The third study did not report fasting GH levels (76). The fourth study observed higher fasting GH levels in TS patients than controls, however this difference was attributed to the fact that over half of the TS group was undergoing estrogen replacement (47). There also were no differences in average 24-hour GH levels or area under the curve for GH, which was sampled through an indwelling needle every 30 minutes for 24h in 10 patients (17). The fifth study reported GH levels in a combined group of individuals with TS and complete gonadal dysgenesis with 46XX karyotype, noting that those with DM had higher levels of plasma GH at the 15 and 30 minute time points; however, the frequency of abnormal GH responses in the diabetic group was not higher than in the non-diabetic group (33).

Two of these studies demonstrated normal rise in GH to hypoglycemic conditions (47, 76). Another pair of studies observed excessive increases in plasma GH levels during hyperglycemic conditions. This dysregulated GH response to hyperglycemia did not correlate with glucose tolerance (47, 75). Thus, while GH secretion to hypoglycemia in TS is intact, GH secretion may be dysregulated with respect to hyperglycemic conditions in individuals with TS. This may be exacerbated by HRT, but it does not seem to be a predominant mechanism underlying hyperglycemia in TS. A review of key GH studies in TS hyperglycemia can be found in **Table 2**.

**Table 2:** Summary of key studies evaluating growth hormone secretion in TS and its potential relationship to the TS-associated hyperglycemia phenotype.

Dysregulated GH secretion	
Costin et al. Carbohydrate intolerance in Gonadal Dysgenesis: Evidence for Insulin Resistance and Hyperglucagonemia. 1985.	Fasting and AUC GH levels were not different between individuals with TS and controls.
Lindsten et al. The Occurrence of Abnormal Insulin and Growth Hormone Responses to Sustained Hyperglycemia in a Disease with Sex Chromosome Aberrations, including a histological study of the pancreas in 2 such patients. 1967.	Fasting GH levels were higher in those with TS, and GH levels did not suppress to hyperglycemia. GH responses were normal to hypoglycemia in those with TS, but GH levels did not suppress to hyperglycemia. However, there was not an increased prevalence of IGT or DM in the group with abnormal GH responses.
Van Campenhout et al. Carbohydrate Tolerance in Gonadal Dysgenesis: 1979.	Glucose tolerance status on OGTT was associated with abnormal GH responses (i.e., lack of suppression) during OGTT, but those with DM had higher fasting GH levels.
Rasio et al. Diabetes Mellitus in Gonadal Dysgenesis. Studies of Insulin and Growth Hormone Secretion. 1976.	No differences in GH responses during OGTT when individuals with TS were divided by glucose tolerance status.

These studies suggest that endogenous GH secretion may be dysregulated in TS, but this does not seem to affect glucose metabolism. GH, growth hormone; TS, Turner syndrome; IGT, impaired glucose tolerance; OGTT, oral glucose tolerance test; DM, diabetes mellitus.

### 6.2 Autoimmunity

A high prevalence of organ-specific autoantibodies was an early phenotypic association made in studies of TS (77, 78). Celiac disease, an autoimmune disorder that is strongly associated with the HLA-DQA1*0501 and DQB1*0201 alleles (79), was subsequently noted to be highly prevalent in TS (80), increasing the plausibility of a type 1 DM (T1D)-like mechanism underlying hyperglycemia in TS. Two early studies (81, 82) assessed human leukocyte antigen (HLA) associations with TS: one demonstrated no skewing of HLA frequencies among those with TS and their parents (82), while the second revealed that the frequencies of B38 HLA class 1 antigens were increased in the TS population (81). One additional study specifically looked for enrichment of HLA DR3 or DR4 haplotypes, two high risk T1D HLA haplotypes (83). These two haplotypes were not identified in those with TS, but they were not studied specifically in the context of TS individuals with a glycemic phenotype (84). An in-depth assessment of T1D genetic risk loci has not been performed in individuals with TS with regards to glycemia.

There is no consensus as to whether the frequency of T1D is increased in TS (85–88). However, most cases of DM in TS do not resemble classic autoimmune DM (58, 85). Longitudinal testing for islet autoantibodies has not been completed in the TS population, so it is not known if indolent autoimmunity contributes to the TS hyperglycemia phenotype. Cross-sectional measurements of islet cell autoantibody (ICA) have been performed but were not identified in any individuals with TS (3, 32, 54, 89, 90). Glutamic acid decarboxylase autoantibody levels (anti-GAD) were measured in 4 studies (32, 54, 91), with a positive assay in 2-10% of individuals with TS. A study of 113 women with TS (3) measured both ICA and anti-GAD antibodies and reported that 2/113 (1.8%) were autoantibody positive but did not differentiate which antibody(s). The time-course of insulin, glucose, and insulinogenic index in response to glycemic challenge did not differ between the antibody-positive and antibody-negative subjects with DM. A study following 134 patients with TS for a mean of 5.4 years similarly demonstrated a prevalence of T1D of 1.5% (92). In contrast to the previous data, in a German study of 24 individuals with TS and DM, 78% were positive for islet autoantibodies (85).

The supply of TS pancreas specimens available for histological study is very limited, and this is reflected in the paucity of research literature on pancreatic pathologies in TS. Reports of only two TS samples have been published, and there was no indication of insulitis or other typical histological findings of type 1 DM (47). However, neither sample was obtained from a TS individual with known DM. Given the natural history of hyperglycemia in TS, it is unlikely that an aggressive T1D-like autoimmune process is the cause of TS hyperglycemia. To evaluate this hypothesis rigorously, it will be necessary to do longitudinal studies that combine glycemic phenotyping and islet autoantibody assessments with genotyping for genetic variants linked to high risk for T1D. The next section (6.3) will discuss evidence suggesting that defects of insulin secretion do exist in TS, making such studies potentially more important to undertake. Key studies related to autoimmunity are summarized in **Table 3**.

**Table 3:** Summary of key studies evaluating autoimmunity as a potential mechanism contributing to TS-associated hyperglycemia.

Autoimmunity	
Dacou-Voutetakis et al. Increased Frequency of Hla B17 Antigen in Girls with Turner syndrome and their Fathers. 1993.	43 girls with TS were compared to 433 controls, with no difference in the frequency of HLA-DR3 or DR4.
Ibarra-Gasparini. New Insights on Diabetes in Turner syndrome: Results from an Observational Study in Adulthood. 2017.	1.8% of TS subjects had positive islet antibodies (14% of those with DM had positive islet antibodies), but those with positive antibodies had similar glycemic phenotyping to those who were antibody negative.
Lindsten et al. The Occurrence of Abnormal Insulin and Growth Hormone Responses to Sustained Hyperglycemia in a Disease with Sex Chromosome Aberrations, including a histological study of the pancreas in 2 such patients. 1967.	2 histological samples were examined, and neither showed evidence of insulitis. One sample demonstrated hyperplastic islet tissue, and those beta cells had significantly smaller nuclei than control samples.

Existing cross-sectional studies and clinical trajectory suggest that a predominant autoimmune mechanism underlying TS-associated hyperglycemia unlikely, but definitive genetic and longitudinal studies are lacking to completely conclude. TS, Turner syndrome; HLA, human leukocyte antigen; DM, diabetes mellitus.

### 6.3 Impaired insulin secretion versus insulin resistance

OGTT-based analyses indicate that insulin secretion is impaired in TS and that this impairment contributes to hyperglycemia. Seven studies utilizing OGTT conclude that insulin secretion is impaired in individuals with TS who have IGT (3, 14, 16, 20, 93–95); several of these studies used age- (16, 20, 94, 95), sex- (16, 20, 94, 95), and BMI-matched (94, 95) controls. Additionally, several other studies have demonstrated that there are defects in insulin secretion even in young, non-obese individuals with TS and NGT as compared to age and sex-matched controls. In one large study (n = 25 women with TS), early defects in insulin secretion were accompanied by heightened insulin sensitivity, suggesting that perhaps glucose intolerance progresses over time in TS as insulin secretory capacity falls and insulin resistance becomes apparent (59).

Abnormalities in insulin secretion are less apparent when assessed *via* intravenous glucose tolerance test (IVGTT) compared to OGTT. Only two of the six IVGTT studies demonstrated any impairment in early insulin secretion (20, 59), with a modest correlation of declining insulin secretion with age (59). Thus, although defects in insulin secretion are sometimes detected by intravenous glucose administration (IVGTT), they are less pronounced than the defects seen by oral glucose administration, i.e., by OGTT.

Insulin secretion in response to non-glucose stimuli has been evaluated in even fewer studies, all with small sample sizes and sometimes lacking control groups. Early phase insulin response to tolbutamide is likely decreased based on results from two studies (75, 96). Insulin secretion in response to glucagon seems to be normal in TS. The peak insulin levels were similar in TS and controls, although with a lower AUC in those with TS (96). Similarly, insulin secretion in response to arginine (17) and to arginine plus GLP-1 also do not appear to be different between individuals with TS and controls (94). The discrepancy in beta-cell capacity in response to different secretagogues is consonant with the fact these stimuli involve different mechanisms of insulin secretion [74] and may provide areas for future investigation with regards to the mechanism of impaired insulin secretion in TS.

Studies evaluating insulin resistance in TS are less consistent than those evaluating insulin secretion, suggesting that insulin resistance is not the primary driver of hyperglycemia in TS. Poor reproducibility of these studies, in part, reflects the variability with respect to methodology, age of TS participants, matching strategies, and medications utilized in the TS group. Five total euglycemic clamp studies have been published in TS (17, 94, 97–99) – two concluded that insulin resistance was increased in TS but did not use adiposity-matched controls (97, 98). Thus, it is possible that excess adiposity is an unmeasured confounder. When individuals with TS are matched on BMI, comparisons of insulin resistance as determined *via* euglycemic clamp are equivocal: one study did not find differences in insulin sensitivity (13 women with TS) (94), whereas the other did find lower insulin sensitivity (4 adolescents with TS) (99). The negative study was matched on fat mass % and lean body mass % as determined by dual-energy X-ray absorptiometry, although the waist-hip ratio was higher in the TS group compared to the controls.

Additional evidence suggesting that insulin resistance is not the primary driver of hyperglycemia is highlighted in three studies comparing IVGTT-derived measures of insulin sensitivity in individuals with TS to controls (20, 59, 94). In all three studies, the subjects were matched on adiposity (either BMI or fat mass % or lean body mass %), and none of the TS patients demonstrated increased insulin resistance. In fact, the largest study (49 individuals with TS) revealed that those with TS had improved insulin sensitivity compared to controls (59). Furthermore, a study utilizing insulin tolerance testing reported that insulin sensitivity was higher in TS (14). Another stream of evidence also suggests that insulin resistance is not driver of early metabolic defects in TS, in that fasting glucose and insulin levels in euglycemic individuals with TS are similar or even lower than in controls (19, 36, 47, 96, 97, 100). OGTT-derived measures of insulin sensitivity also consistently demonstrate comparable insulin sensitivity in normoglycemic, non-obese TS women and controls (32, 59, 95, 101). Finally, to confirm the central obesity seen by abdominal magnetic resonance imaging, TS individuals underwent biochemical testing. All of them had the biochemical hallmarks of central adiposity but not of hyperinsulinemia (36).

Thus, based on multiple lines of evidence, insulin secretion in response to oral glucose seems to be diminished and may decline with age in TS. The insulin secretory responses to IV stimuli, including glucose, arginine, glucagon, and GLP-1 are less clearcut and warrant additional study with larger sample sizes and better matching. Insulin resistance does not appear to contribute consistently to early hyperglycemia in TS. It is likely that insulin resistance becomes evident as central adiposity increases in TS, and that this exacerbates the pre-existing defect in insulin secretion. Key studies summarized in this section can be found in **Tables 4** and **5**.

**Table 4:** Summary of key studies evaluating insulin secretion as a potential mechanism contributing to TS-associated hyperglycemia in.

Impaired insulin secretion	
Bakalov et al. Impaired Insulin Secretion in the Turner Metabolic Syndrome. 2004.	HOMA-B, I-AUC_30,_ I-AUC_180_, and FPIR derived from OGTT were all significantly reduced in subjects with TS compared to controls (who were age and BMI matched women with primary ovarian failure). Subgroup analysis of the TS subjects with NGT continued to demonstrate lower insulin responses.
Hjerrild et al. Delayed Beta-Cell Response and Glucose Intolerance in Young Women with Turner Syndrome. 2011.	I-AUC_120_ and I:G derived from OGTT were significantly reduced in subjects with TS compared to controls. FPIR derived from IVGTT was also reduced in subjects with TS.
Sheanon et al. Increased Prevalence of Beta-Cell Dysfunction Despite Normal HbA1c in Youth and Young Adults with Turner Syndrome. 2021.	Oral c-peptide and glucose minimal model-derived indices of beta cell function were significantly reduced in subjects with TS compared to controls.

These studies demonstrate that insulin secretion is impaired to oral glucose and is present prior to the evidence of hyperglycemia. TS, Turner syndrome; HOMA-B, Homeostatic model assessment of beta cell function; I-AUC, area under the insulin curve; FPIR, first phase insulin response; OGTT, oral glucose tolerance test; TS, Turner syndrome; BMI, body mass index; NGT, normal glucose tolerance; I:G; insulin-to-glucose ratio; IVGTT, intravenous glucose tolerance test.

**Table 5:** Summary of key studies evaluating insulin resistance as a contributor to TS-associated hyperglycemia.

Insulin resistance	
Caprio et al. Insulin Resistance: An Early Metabolic Defect of Turner’s Syndrome. 1991.	Individuals with TS required significantly less GIR to maintain euglycemia than controls.
Hjerrild et al. Delayed Beta-Cell Response and Glucose Intolerance in Young Women with Turner Syndrome. 2011.	Insulin-stimulated glucose uptake was not different between individuals with TS and controls.
O’Gorman et al. An Evaluation of Early Cardiometabolic Risk Factors in Children and Adolescents with Turner syndrome. 2013.	WBISI (calculated using Matsuda index) from OGTT was not different between subjects with TS and controls. BMI and waist circumference were not correlated with WBISI in the TS group.
Alvarez-Nava et al. Insulin Sensitivity and Pancreatic B-Cell Function in Ecuadorian Women with Turner Syndrome. 2020.	HOMA-IR, Matsuda Index, QUICKI were not different between subjects with TS and controls.

These studies demonstrate that insulin resistance does not contribute consistently to early glucose abnormalities in TS. TS, Turner syndrome; GIR, glucose infusion rate; WBISI, whole-body insulin sensitivity; OGTT, oral glucose tolerance test; BMI, body mass index; HOMA-IR, homeostatic model assessment of Insulin Resistance; QUICKI, Quantitative Insulin Sensitivity Check Index.

### 6.4 Hyperglucagonemia/dysregulated glucagon

Glucagon is a key counterregulatory hormone to insulin and has been hypothesized to play a role in the diabetogenic phenotype (102). Three studies, all published prior to 1991, investigated glucagon behavior in individuals with TS (17, 99, 103). All of them noted either normal or no differences in fasting glucagon levels. The third study also noted significantly higher glucagon levels in the individuals with TS following OGTT as well as diminished levels during insulin-induced hypoglycemia. These results may indicate abnormal glucagon secretion in TS but should be interpreted with caution as the controls in this study were not sex- or adiposity-matched. Synthesizing the limited data available, glucagon secretion may be dysregulated in TS, but there is insufficient evidence to say that hyperglucagonemia plays a predominant role in the heightened risk for hyperglycemia in TS. These studies are summarized in **Table 6**.

**Table 6:** Summary of key studies evaluating hyperglucagonemia as a potential mechanism contributing to TS-associated hyperglycemia.

Hyperglucagonemia	
Zinman et al. Endocrine, Cytogenetic, and Psychometric features of patients with X-isochromosome 46,X,i(Xq) Turner’s syndrome: A Preliminary Study in Nine Patients. 1984.	Fasting glucagon levels were reported as normal when compared to previously-studied controls, and did not change during OGTT.
Costin et al. Carbohydrate Intolerance in Gonadal Dysgenesis: Evidence for Insulin Resistance and Hyperglucagonemia. 1985.	No difference in fasting glucagon levels between individuals with TS and controls, but glucagon was higher at all time points throughout an OGTT in those with TS.
Stoppoloni et al. Characteristics of Insulin Resistance in Turner syndrome. 1990.	No differences in fasting glucagon levels between individuals with TS and controls.

The 3 small studies show no differences in fasting glucagon levels between individuals with TS and controls, but limited data is available to conclude if stimulated glucagon secretion is dysregulated. TS, Turner syndrome; OGTT, oral glucose tolerance test.

### 6.5 Impaired incretin secretion

As discussed in section 6.3, the early studies of hyperglycemia in TS reported disparate responses to intravenous and oral glucose challenges (15, 75). This implicates incretin hormones as possible contributors to hyperglycemia in TS. The incretin hormones, including glucose-dependent insulinotropic polypeptide (GIP) and glucagon-like 1 peptide (GLP-1), are secreted by enteroendocrine cells in response to ingested macronutrients. The incretins potentiate insulin secretion in a glucose-dependent manner (104). OGTT followed by oral amino acid tolerance tests conducted in 20 individuals with TS demonstrated that those individuals with IGT detected on OGTT had similar plasma glucose and insulin responses to oral amino acids compared to those with NGT (75). These results suggest that individuals with TS and abnormal glucose tolerance can have normal tolerance and normal beta-cell sensitivity to oral amino acids and thereby provides further indirect evidence that alterations in incretin secretion may underly hyperglycemia in TS. Only two studies have directly measured incretin hormones during OGTT and contrast with each other. A small, early study of 12 adolescents with TS showed abnormal GIP response during the OGTT in those with IGT, and this impairment correlated with delayed insulin secretion (93). The other published study directly evaluating incretin concentrations in TS demonstrated no significant differences in serum GIP and GLP-1 levels between 13 individuals with TS and age-, sex-, BMI-, and lean body mass matched controls during OGTT, although notably only 2 women with TS had hyperglycemia (94). No TS studies have directly quantified the incretin effect in TS using isoglycemic IV glucose infusion. This is an area that warrants future study, as GLP-1 agonists are gaining traction to treat hyperglycemia in other conditions with high DM risk (105). Key studies discussed here are summarized in **Table 7**.

**Table 7:** Summary of studies evaluating impairment in incretin secretion or action to underly the TS-associated hyperglycemia phenotype.

Impaired incretin secretion or action	
Polychronakos et al. Carbohydrate Intolerance in Children and Adolescents with Turner syndrome. 1980.	Paired OGTT and IVGTT in TS showed that subjects with TS tolerated IV glucose infusion better than oral glucose (i.e., the mean insulin, glucose and Kt values were not different between those with normal oral glucose tolerance and abnormal oral glucose tolerance.
Heinze et al. Reduced Secretion of Gastric Inhibitory Polypeptide in Turner Patients with Impaired Glucose Tolerance. 1991.	Adolescents with TS had abnormal GIP response during the OGTT in those with IGT, and this impairment correlated with delayed insulin secretion.
Hjerrild et al. Delayed Beta-Cell Response and Glucose Intolerance in Young Women with Turner Syndrome. 2011.	No significant differences in serum GIP and GLP-1 levels between 13 individuals with TS and age-, sex-, BMI-, and lean body mass matched controls during OGTT

OGTT, oral glucose tolerance test; IVGTT, intravenous glucose tolerance test; TS, Turner syndrome, Kt, total glucose disappearance rate; GIP, glucose-dependent insulinotropic peptide; IGT, impaired glucose tolerance; GLP-1, glucagon-like peptide 1; BMI, body mass index.

## 7 Clinical considerations

### 7.1 Recommended screening tests and potential limitations

The Clinical practice guidelines for the care of girls and women with TS recommend annual screening for DM after the age of 10 with HbA1c, with or without fasting plasma glucose (2). However, studies including HbA1c in TS are limited and show discordance between HbA1c and OGTT in the diagnosis of IGT and DM according to American Diabetes Association criteria in adults (3, 18, 106). It has similarly been demonstrated that 23% of girls with TS had abnormal OGTTs despite normal HbA1c (54). Isolated fasting glucose intolerance is uncommon in TS (14, 32, 35, 95) and paradoxically, a reduction of fasting insulin and plasma glucose concentrations has been associated with a deterioration in glucose tolerance in TS (20). As such, serial OGTT may be a more sensitive means to screen for glucose abnormalities in TS. A 2011 study demonstrated that a significant percentage of adult patients with TS lacked medical follow up, and those without transition care or preceding specialist care had an increased risk of having undiagnosed cardiovascular risk factor (107). Thus, it is crucial that patients with TS understand the necessity of and differences in metabolic screening as they transition to adult care.

### 7.2 Treatment considerations

There are no TS-specific treatment guidelines for DM. However, optimization of DM treatment should be a priority given the significant contribution of diabetes to morbidity and mortality in TS. Additionally, DM is associated with lower health-related quality of life in TS (108) making this an important area of research. There are no published studies reporting the types of DM treatments used in TS and how they vary by age and in different health care settings. There are also no studies reporting the efficacy of targeted lifestyle modifications or anti-diabetic agents to mitigate the progression from IGT to DM in TS – given the natural history of hyperglycemia in TS (3), this should be an area of priority. It could be that most individuals with TS and DM are started on metformin in line with American Diabetes Association guidelines for the pharmacologic treatment of type 2 DM (109). However, given that insulin secretory defects seem to exist early on, even before the development of glucose abnormalities, and fasting hyperglycemia is atypical in TS, this drug may be a less appropriate first-line hyperglycemia treatment for individuals with TS.

## 8 Conclusion

Hyperglycemia is common in TS and is probably caused, at least in part, by an intrinsic defect in insulin secretion. Additional physiological studies are needed to clarify the mechanisms underlying hyperglycemia in TS. Hopefully, these efforts will culminate in physiology-informed therapy to prevent and/or treat hyperglycemia in TS patients and further inform screening for hyperglycemia in TS. The substantial increase in mortality associated with DM in TS is unexplained and warrants further study as these statistics are based on retrospective review of death certificate data and thus relevant clinical data is lacking. Future studies need to be designed with enough statistical power to account for the heterogeneity within the TS population, and this may be aided by TS registries and research networks (110). Genetics studies should continue to be pursued, as the specific X_chr_ contributions to DM in TS remain elusive.

## Author contributions

CTP conceptualized the article. CTP and CM performed the literature search. CM, CTP, and EA wrote the first draft of the article. CTP and AWN critically revised the work. All contributing authors have read and approved submission to Frontiers in Pediatric Endocrinology. All authors contributed to the article and approved the submitted version.
